# Are Volumetric Bone Mineral Density and Bone Micro-Architecture Associated with Leptin and Soluble Leptin Receptor Levels in Adolescent Idiopathic Scoliosis? – A Case-Control Study

**DOI:** 10.1371/journal.pone.0087939

**Published:** 2014-02-06

**Authors:** Elisa M. S. Tam, Fiona W. P. Yu, Vivian W. Y. Hung, Zhen Liu, King Lok Liu, Bobby K. W. Ng, Simon K. M. Lee, Yong Qiu, Jack C. Y. Cheng, Tsz-Ping Lam

**Affiliations:** 1 Department of Orthopaedics & Traumatology, The Chinese University of Hong Kong, Hong Kong, China; 2 Lee Hysan Clinical Research Laboratory, The Chinese University of Hong Kong, Hong Kong, China; 3 Spine Surgery, The Affiliated Drum Tower Hospital of Nanjing University Medical School, Nanjing, China; 4 Joint Scoliosis Research Center of the Chinese University of Hong Kong and Nanjing University, Hong Kong, China; UCSD School of Medicine, United States of America

## Abstract

**Background:**

Adolescent idiopathic scoliosis (AIS) is associated with low bone mineral density (BMD). The underlying etiology and how it may relate to the development of osteopenia remains unknown. Leptin has been postulated as one of the etiologic factors of AIS because of its profound effects on bone metabolism and pubertal growth. Its modulator, soluble leptin receptor (sOB-R), may affect leptin bioavailability and signaling. This study aimed to investigate whether serum leptin and sOB-R levels may be associated with bone quality, and whether these relationships may differ between young adolescent girls with and without AIS.

**Methods:**

This was a case-control study involving 94 newly diagnosed AIS girls (Cobb angle 12–48°) aged 12 to 14 years old and 87 age and gender-matched normal controls. Subjects with BMI>23.0 Kg/m^2^ were excluded. Anthropometric measurements including body weight, height, arm span and sitting height were taken. Serum total leptin and sOB-R were assayed with ELISA. Non-dominant distal radius was scanned with High Resolution pQCT for assessing bone quality in terms of bone morphometry, volumetric BMD (vBMD) and trabecular bone micro-architecture.

**Results:**

Compared with normal controls, AIS girls had numerically higher sOB-R (p = 0.006), lower average vBMD (p = 0.048), lower cortical vBMD (p = 0.029), higher cortical bone perimeter (p = 0.014) and higher trabecular area (p = 0.027), but none remained statistically significant after the Hochberg-Benjamini procedure. Correlation analysis on serum leptin level indicated that distinctive correlations with trabecular bone parameters occurred only in AIS.

**Conclusion:**

This study showed that bone quality in AIS girls was deranged as compared with controls. In addition, the distinct differences in correlation pattern between leptin and trabecular bone parameters indicated possible abnormalities in bone metabolism and dysfunction of the leptin signaling pathway in AIS.

## Introduction

Adolescent Idiopathic Scoliosis (AIS) is a complex three-dimensional spinal deformity affecting mainly girls with disease onset at the peripubertal period [1], [2]. AIS can be a serious condition carrying significant morbidities especially for patients with severe curves. It is important to elucidate the etiopathogenesis of AIS so that effective preventive and therapeutic measures can be devised. Previous studies have reported the association between AIS and low body weight, tall stature, increased arm span, low body mass index, delayed menarche and low bone mass [3], [4], [5], [6], [7], [8], [9], [10], [11], [12], [13], [14], [15]. Special attention is paid to low bone mass as Hung *et al.* [16] showed that it was a significant prognostic indicator for curve progression. What mediates these observed characteristic anthropometric phenotypes, delayed pubertal development, abnormal skeletal growth and disturbed bone mineral homeostasis in AIS remains unresolved.

Leptin, a 16 kDa protein hormone predominantly secreted by adipocytes, is known to be an important factor in neuro-osseous development affecting skeletal growth, the onset of puberty, energy expenditure and body composition [17], [18], [19], [20], [21], [22], [23]. Leptin actions are mediated through leptin receptors expressed in the hypothalamus and peripheral tissues [24], [25], [26], [27], [28], [29], [30]. The peripheral effects of leptin have been shown to be directly anabolic to bone formation [31], promoting the proliferation of osteoblasts [32] and chondrocytes [33], stimulating osteoblastic differentiation [32], [34], mineralization [29], [32], [35] and inhibiting osteoclastogenesis and osteoclast activity [32], [36], [37]. On the other hand, leptin have also been shown to exert a catabolic effect on bone formation through the hypothalamus via the sympathetic nervous system (SNS) [38], [39], [40], [41].

AIS and higher levels of serum leptin are found more commonly in girls versus boys, suggesting that leptin may play an important role in the pubertal growth of girls [42], [43]. The abnormal phenotypic profiles that characterize AIS girls including low body weight, tall stature, increased arm span, low body mass index, delayed menarche and low bone mass also happen to coincide with leptin’s physiological effects [3], [4], [5], [6], [7], [8], [9], [10], [11], [12], [13], [14], [15]. It was thus logical to investigate the possible role of leptin in the etiopathogenesis of AIS. A previous study [44] has shown that children with lower BMI have more severe truncal asymmetry. Girls with lower BMI likely have lower leptin levels that could be a potential cause of AIS. In mice, Wu *et al*. [45] reported that high central leptin activity might increase the risk of developing scoliosis. In a previous study conducted by our joint center, significantly lower serum leptin levels were found in AIS girls without controlling for the BMI status [46]. Subsequent studies by our group documented increased serum levels of sOB-R in AIS as compared with matched normal controls [15]. It is possible that changes in the sOB-R level might alter the bioavailability of leptin at the cellular and tissue levels, resulting in the abnormal phenotypic expression that was observed in AIS girls.

To the best of our knowledge, bone quality assessment with HR-pQCT and its correlation with leptin bioavailability has not been reported in the literature. The purpose of the current study aimed at testing the hypothesis that 3-dimensional bone quality parameters and its correlations with serum total leptin and sOB-R levels are distinct in AIS when compared with normal matched controls.

## Materials and Methods

### Ethics Statement

Ethics approval for the present study was obtained from the Joint Chinese University of Hong Kong - New Territories East Cluster Clinical Research Ethics Committee (CREC), Hong Kong (CRE 2010.066). Informed written consents were obtained from all the subjects as well as from their legal guardians before enrollment into the study.

### Subjects Recruitment

94 Chinese AIS girls aged between 12 and 14 years old without prior treatment were recruited consecutively from our scoliosis clinic. 87 age-matched healthy girls were recruited from local schools. Subjects with BMI>23.0 were excluded to avoid bias from the relatively high serum leptin levels known to be present in overweight/obese subjects [47], [48]. The diagnosis for AIS (Cobb angle ≥10°) was confirmed with detailed clinical and radiological assessment and the standard standing antero-posterior radiographs were used to assess curve severity. Curve types of the AIS girls were recorded according to the Lenke Classification [49]. All control subjects were examined by an experienced orthopaedic surgeon (Dr. T.P. Lam) to rule out spinal deformities. Subjects with history of congenital deformities, neuromuscular diseases, skeletal dysplasia, endocrine diseases, connective tissue abnormalities, mental retardation, or history of recent steroid intake were excluded from the study.

### Anthropometric Assessment

Anthropometric parameters including body height, body weight, sitting height and arm span were measured. Height was recorded without shoes, standing against a wall-mounted stadiometer and measurement was taken to the nearest 0.1 cm. Weight was measured to the nearest 0.1 kg in light clothes without shoes. Sitting height was measured by a stadiometer, the subjects were asked to sit straight and measurement was taken to the nearest 0.1 cm. Arm span was measured by using a wall-mounted tape to the nearest 0.1 cm. As shown by previous studies [50], [51], the different equations for correction of height loss resulted from the spinal deformity had limitation and might not fit every curve. Moreover, it has been shown that linear correlation between arm span and standing height in healthy children and adolescents is very high (r^2^ = 0.99) [52]. Hence, arm span was used for calculating the BMI in place of body height in the current study (body weight/armspan^2^).

### Assessment of Bone Quality with High Resolution pQCT

**Figure 1:**
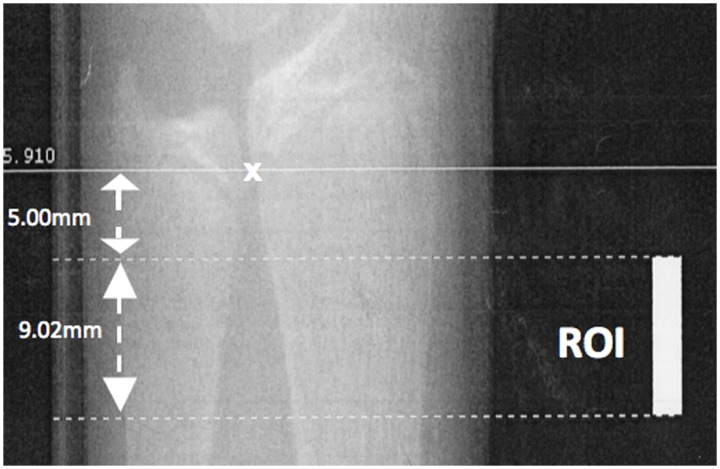
Representative scout view of the distal radius scanned with high resolution peripheral quantitative computed tomography. The reference line is marked at the most proximal point of the inner aspect of the growth plate (marked with x). The region of interest (ROI) spanning 9.02 mm starts from 5 mm proximal to the reference line. 110 CT slices with a nominal resolution (voxel size) of 82 µm were obtained.

The non-dominant distal radius was measured by HR-pQCT (XtremeCT, Scanco Medical, Brüttisellen, Switzerland) according to the standard protocol [53]. Subjects were instructed to put their forearm in a customized cast and hold the handle of the cast to minimize the variation of rotation between and within subjects. Using a scout view, a reference line was placed at the most proximal limit of the inner aspect of the epiphyseal growth plate as shown in Figure 1. The scan was performed on a segment spanning 9.02 mm starting from 5 mm proximal to the reference line. 110 CT slices with a nominal resolution (voxel size) of 82 µm were obtained using the following settings: a microfocus X-Ray-source of 60 kVp and a matrix size of 1536×1536. The effective dose was less than 5 µSv and the measurement time was 3 minutes. Distal radius measurements using HR-pQCT were found to correlate well with systemic BMD status in premenopausal women [54], and combined with its low radiation and avoidance of weight bearing effect on BMD, it represented a favorable bone imaging technique for our adolescent subjects.

**Figure 2:**
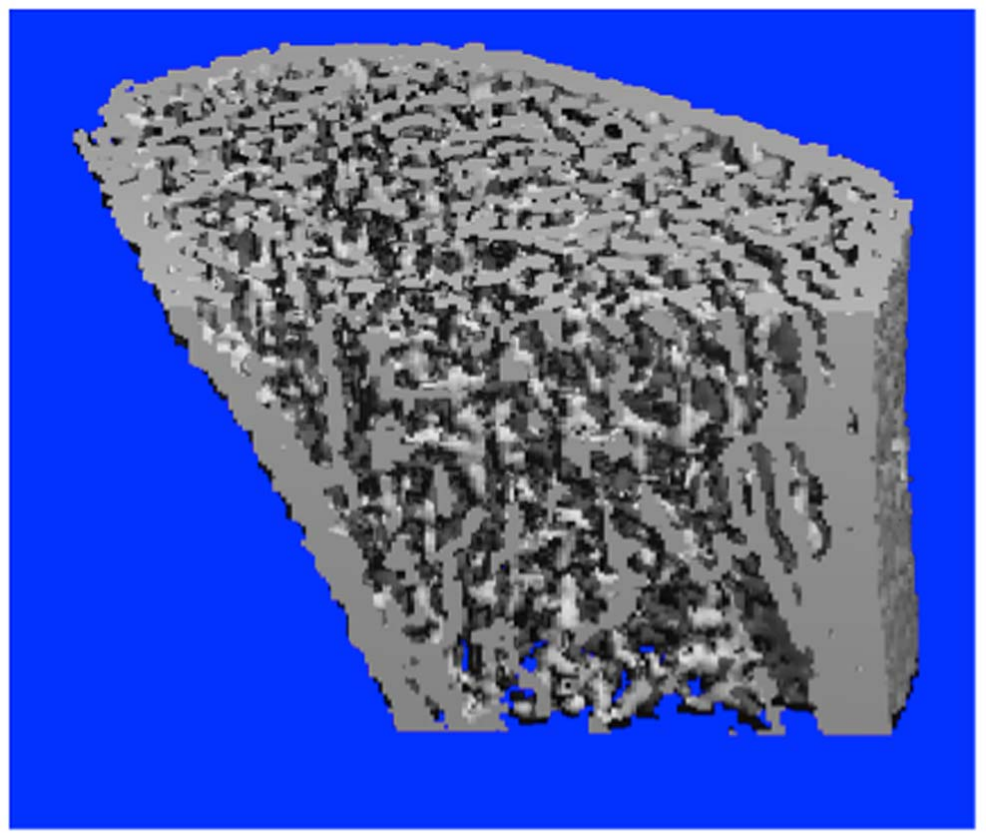
Three-dimensional HR-pQCT image of the distal radius with crosscut showing the trabecular bone.

Methods used to process HR-pQCT data have been described in details by Laib *et al.* [55]. A typical reconstructed image was shown in Figure 2. HR-pQCT bone parameters were divided into three categories namely A) volumetric bone mineral density (vBMD), B) bone morphometry, and C) trabecular bone micro-architecture.

#### A) Volumetric BMD

Average vBMD, Cortical Bone vBMD and Trabecular Bone vBMD in milligram of hydroxyapatite per cubic centimeter (mgHA/cm^3^) were computed as the average mineral density of the total, cortical and trabecular volume of interest, respectively. The CV% for various vBMDs ranged from 1.27% to 2.21% for the study population.

#### B) Bone morphometry

**Figure 3:**
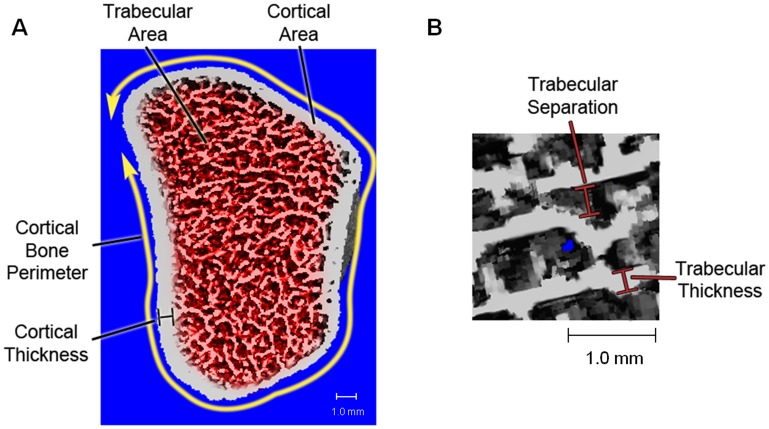
Illustrations of A) bone morphometry parameters and B) trabecular separation and thickness.

The focused zone was automatically separated into cortical and trabecular compartments using a Gaussian filter and a threshold-based algorithm. The threshold used to discriminate the cortical from trabecular bone was set to one third of the apparent cortical bone density as previously described by Laib *et al.* [55]. Trabecular Area and Cortical Area were measured directly. Cortical Bone Perimeter was calculated from the contour. Mean Cortical Thickness was defined as the mean cortical volume divided by the outer bone surface. Illustration of these parameters is shown in Figure 3A. Excellent correlation (r = 0.98) has been shown for Cortical Thickness measurement compared with the gold standard of ex-vivo microCT data [56]. The coefficient of variation % (CV%) ranged from 1.35%–6.58%.

#### C) Trabecular bone micro-architecture

Trabecular Bone Volume to Tissue Volume Ratio (BV/TV) was derived from Trabecular Bone vBMD assuming mineral density of fully mineralized bone of 1200 mg hydroxyapatite [i.e. Trabecular Bone vBMD (mgHA/cm^3^)/1200 mgHA/cm^3^]. The spatial resolution of the resulting images from HR-pQCT is not sufficient to depict individual trabeculae, micro-architecture parameters were extracted with the help of a ridge detection algorithm described previously by Laib *et al*. [55], [57] and Hildebrand and Rüegsegger [58]. Trabecular Number (mm^−1^) was defined as the inverse of the mean spacing of the ridges [55], [57] and thus a truly three-dimensional data is evaluated independent of the plate or rod-like nature of the trabeculae structure. Trabecular Thickness (mm) and Trabecular Separation (mm) were derived from BV/TV and Trabecular Number using standard histomorphometry and by the following formulae: Trabecular Thickness = (BV/TV)/Trabecular Number and Trabecular Separation = (1-BV/TV)/Trabecular Number. Illustration of the physical meanings of these parameters is shown in Figure 3B. In the validation studies, excellent correlations (r≥0.96) have been shown for the values of these trabecular bone micro-architecture parameters when compared with the microCT measurements [59]. The CV% for the various parameters ranged from 1.25% to 4.65%.

### Biochemical Assays

Non-fasting peripheral venous blood samples were collected from the subjects, serum was separated and stored at −80°C until analysis. Serum total leptin levels were measured by enzyme-linked immunosorbent assay (ELISA) specific for human leptin (ALPCO Diagnostics, Salem, NH, USA) following a standard protocol. The lowest detection limit for leptin was 0.50 ng/ml. Serum sOB-R levels were measured by ELISA (R&D systems, Minneapolis, USA). The minimum detectable concentration was 0.057 ng/ml.

### Definition of Terms

For sake of clarity, terminology on leptin presented in this report is defined as follows:

**Soluble leptin receptor (sOB-R)** – main leptin binding protein in circulation, not a signalling receptor

**Leptin receptor (Ob-R)** – membrane bound signalling/transport receptor for free leptin

**Free leptin** – active form of leptin that could be bound to leptin receptor

**Bound leptin** – leptin that is reversibly bound to sOB-R in the circulation

**Serum total leptin** – free leptin+bound leptin in serum

**Leptin bioavailability** – amount of leptin that is available to leptin receptor on target tissues for signal transduction

### Statistical Analysis

Data are presented as mean ± SD. Anthropometric data, bone quality data, serum total leptin levels, and sOB-R levels were compared between AIS and normal controls using independent samples *t*-test. Bone quality measurement by HR-pQCT was separated into cortical bone parameters (cortical vBMD, cortical area, cortical thickness, and cortical bone perimeter) and trabecular bone parameters (trabecular vBMD, trabecular area, trabecular BV/TV, trabecular number, trabecular thickness, and trabecular separation) for correlation analysis. Multiple regression analysis was used to analyze differences in bone quality parameters between groups with adjustment of age and BMI. The correlations between bone quality parameters and serum total leptin and sOB-R of the AIS and control group were analyzed with Pearson’s correlation analysis and multiple regression analysis with age and BMI adjustment. To correct for multiple comparisons, false discovery rate (FDR) adjusted p-value derived from the Hochberg-Benjamini procedure was determined. SPSS 16.0 for Windows (SPSS Inc., Chicago, IL) was used for statistical analysis. FDR-adjusted p<0.05 was considered statistically significant.

## Results

**Figure 4:**
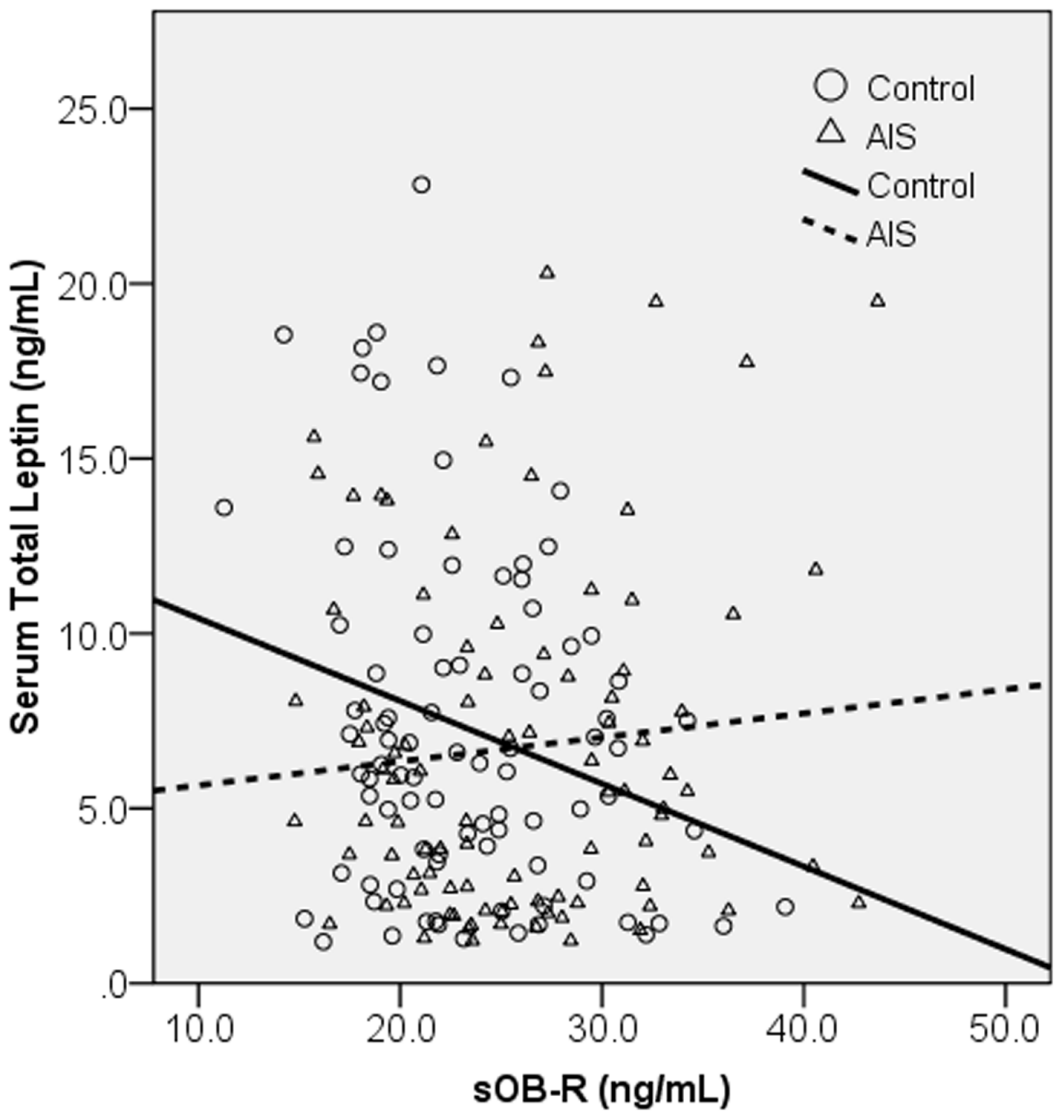
Correlation between serum total leptin and soluble leptin receptor (sOB-R) levels in AIS and controls. Controls showed a significant negative correlation between serum total leptin and sOB-R (r = −0.250, p = 0.020), while AIS showed no correlation (r = 0.089, p = 0.394).

The mean Cobb angle of AIS girls was 25.6±6.7° (range 12–48°). 97.9% of the AIS girls were right handed. According to Lenke Classification, 17.2% of the AIS girls were type 1, 4.3% type 2, 36.6% type 3, 6.5% type 4, 19.4% type 5, and 16.1% type 6. A total of 51.1% of the AIS girls have right thoracic curve component. AIS girls had lower BMI (17.91±2.20 vs. 18.64±2.15 kg/m^2^, p = 0.024), and higher sOB-R (25.86±6.49 vs. 23.43±5.27 ng/mL, p = 0.006) than normal controls (Table 1). Normality test with normal probability plot showed that serum total leptin and sOB-R were fairly normally distributed in both AIS and control groups (results not shown). Age, body weight, body height, arm span, sitting height, tanner stages and serum total leptin were similar between the two groups (Table 1). Controls showed a statistically significant negative correlation between serum total leptin and sOB-R (r = −0.250, p = 0.020), while the correlation could not be detected for AIS subjects (r = 0.089, p = 0.394) (Figure 4).

**Table 1:** Anthropometrics, serum total leptin and sOB-R levels in controls and AIS girls.

	Controls		AIS			
	Mean	SD	Mean	SD	% Difference	p-value
Age	12.93	0.43	13.05	0.52	0.96	0.081
Weight (kg)	43.79	6.71	42.94	6.42	−1.93	0.387
Height (cm)	154.58	6.64	155.24	5.62	0.43	0.472
Arm Span (cm)	153.01	7.43	154.72	6.70	1.12	0.105
Sitting Height (cm)	82.04	3.67	82.30	3.56	0.32	0.638
BMI (Kg/m^2^)**	18.64	2.15	17.91	2.20	−3.92	0.024*
Tanner Stage (breast)	3.02	0.71	3.14	0.80	3.97	0.309
Tanner Stage (pubic hair)	2.45	0.83	2.52	0.77	2.86	0.541
Leptin (ng/mL)	7.26	4.99	6.75	5.01	−7.03	0.493
sOB-R (ng/mL)	23.43	5.27	25.86	6.49	10.37	0.006*

*p-value <0.05.

**BMI by arm span (body weight/armspan^2^).

Abbreviations: AIS, adolescent idiopathic scoliosis; sOB-R, soluble leptin receptor.

AIS girls had numerically lower average vBMD (256.2±51.4 vs. 270.9±45.6 mgHA/cm^3^, age and BMI adjusted p = 0.048, FDR-adjusted p = 0.132), and numerically lower cortical vBMD (688.4±79.0 vs. 712.6±62.5 mgHA/cm^3^, age and BMI adjusted p = 0.029, FDR-adjusted p = 0.106). Trabecular vBMD was numerically lower in AIS than in controls, although the difference did not reach statistical significance (Table 2). In bone morphometry, AIS girls have numerically higher cortical bone perimeter (55.03±4.14 vs. 53.56±4.50 mm, age and BMI adjusted p = 0.014, FDR-adjusted p = 0.106), and numerically higher trabecular area (149.1±27.5 vs. 140.0±27.3 mm^2^, age and BMI adjusted p = 0.027, FDR-adjusted p = 0.106). Trabecular BV/TV, trabecular number and trabecular thickness were numerically lower and trabecular separation was numerically higher in AIS, although the differences did not reach statistical significance (Table 2).

**Table 2:** Comparison of bone quality parameters between controls and AIS.

	Controls		AIS				
	Mean	SD	Mean	SD	% Diff	p-value	Age & BMI adjusted p-value
**A) Volumetric BMD**							
Average Bone Mineral Density (mgHA/cm^3^)	270.9	45.6	256.2	51.4	−5.43	0.044*	0.048*
Cortical Bone Mineral Density (mgHA/cm^3^)	712.6	62.5	688.4	79.0	−3.39	0.023*	0.029*
Trabecular Bone Mineral Density (mgHA/cm^3^)	154.1	26.7	149.0	25.7	−3.31	0.193	0.292
**B) Bone Morphometry**							
Cortical Area (mm^2^)	28.1	10.7	25.8	12.3	−8.47	0.168	0.319
Cortical Thickness (mm)	0.53	0.20	0.47	0.23	−10.22	0.091	0.178
Cortical Bone Perimeter (mm)	53.56	4.50	55.03	4.14	2.74	0.024*	0.014*
Trabecular Area (mm^2^)	140.0	27.3	149.1	27.5	6.52	0.026*	0.027*
**C) Trabecular Bone Micro-architecture**							
Trabecular BV/TV	0.128	0.022	0.124	0.021	−3.25	0.200	0.303
Trabecular Number (mm^−1^)	1.766	0.225	1.728	0.203	−2.18	0.228	0.360
Trabecular Thickness (mm)	0.073	0.007	0.072	0.009	−0.90	0.599	0.625
Trabecular Separation (mm)	0.503	0.074	0.515	0.071	2.44	0.256	0.398

* p-value <0.05.

Abbreviations: AIS, adolescent idiopathic scoliosis; BMD, bone mineral density; BV/TV, bone volume to tissue volume ratio; Diff, difference; HA, hydroxyapatite.

Results of correlation analysis with cortical bone parameters were shown in Table 3. With Pearson’s correlation analysis, controls had significant correlations between serum total leptin and all cortical bone parameters (cortical vBMD (r = 0.287), cortical area (r = 0.294), cortical thickness (r = 0.249) and cortical bone perimeter (r = 0.261)), none of the correlation remained significant after adjustment for age and BMI; while AIS had significant correlations between serum total leptin and cortical vBMD (r = 0.362), cortical area (r = 0.367), and cortical thickness (r = 0.385) after the Hochberg-Benjamini procedure, however, none of the correlation remained significant after adjustment for age and BMI (Table 3). For sOB-R, controls had significant correlations with cortical vBMD (r = −0.229), cortical area (r = −0.248), and cortical thickness (r = −0.239) after the Hochberg-Benjamini procedure, and again none of the correlations remained significant after adjustment for age and BMI; while AIS had no significant correlation (Table 3).

**Table 3:** Correlations between cortical bone parameters, serum total leptin and sOB-R levels with and without age and BMI adjustment.

		Serum Total Leptin		sOB-R	
		Controls	AIS	Controls	AIS
**A)Volumetric BMD**					
Cortical vBMD	r	0.287** #	0.362** #	−0.229* #	−0.116
	β	0.054	0.042	−0.144	−0.800
**B) Bone Morphometry**					
Cortical Area	r	0.294** #	0.367** #	−0.248* #	−0.141
	β	0.041	0.048	−0.147	−0.091
Cortical Thickness	r	0.249* #	0.385** #	−0.239* #	−0.134
	β	0.028	0.081	−0.158	−0.083
Cortical Bone Perimeter	r	0.261* #	−0.146	−0.173	−0.130
	β	0.002	−0.224	−0.051	−0.141

*correlation with p-value <0.05,

**correlation with p-value <0.01,

# statistically significant after Hochberg-Benjamini correction for multiple comparison.

Abbreviations: AIS, adolescent idiopathic scoliosis; β, standardized coefficient calculated by multiple regression analysis with age and BMI adjustment; r, Pearson’s correlation coefficient; sOB-R, soluble leptin receptor; vBMD, volumetric bone mineral density.

**Figure 5:**
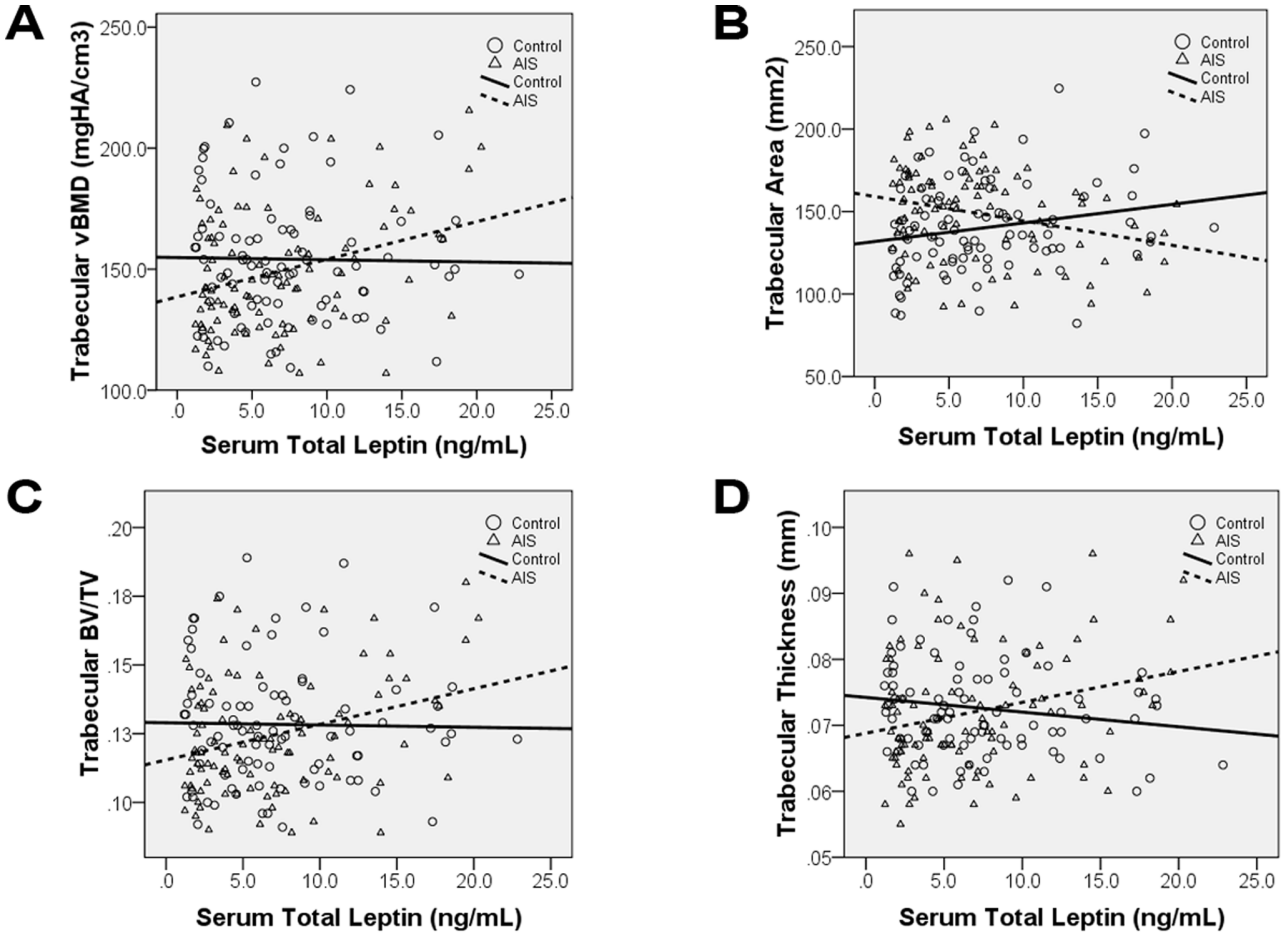
Correlations of various HR-pQCT parameters with serum total leptin in AIS and controls. Correlation analysis showed there was statistically significant correlation between serum total leptin and the following HR-pQCT parameters only in AIS: A) Trabecular volumetric bone mineral density (vBMD), B)Trabecular Area, C) Trabecular bone volume to tissue volume ratio (BV/TV), and D) Trabecular Thickness. The difference in correlations could be observed by crossing of the best fit lines.

Results of correlation analysis with trabecular bone parameters were shown in Table 4. For serum total leptin, AIS showed distinctive correlation pattern that was absent in controls. With Pearson’s correlation analysis, AIS had significant correlations with trabecular vBMD (r = 0.303), trabecular area (r = −0.269), trabecular BV/TV (r = 0.303), and trabecular thickness (r = 0.254) after the Hochberg-Benjamini procedure, all of these correlations remained significant after age and BMI adjustment. In addition, trabecular number (β = 0.265) and trabecular separation (β = −0.262) became significant after age and BMI adjustment. Scatter plots of some of these correlations were shown in Figure 5 to illustrate the difference observed between AIS and control groups. It was evident that the AIS group showed increasing trend in the scatter plots with trabecular vBMD (A), trabecular BV/TV (C), trabecular thickness (D), and decreasing trend in trabecular area (B) versus serum total leptin. Controls, however, showed no correlation in the trabecular vBMD (A), trabecular BV/TV (C), and trabecular thickness (D) plots. For correlations between sOB-R and trabecular bone parameters, both AIS and controls showed no significant correlations (Table 4).

**Table 4:** Correlations between trabecular bone parameters, serum total leptin, and sOB-R levels with and without age and BMI adjustment.

		Serum Total Leptin		sOB-R	
		Controls	AIS	Controls	AIS
**A)Volumetric BMD**					
Trabecular vBMD	r	−0.017	0.303** #	−0.085	0.171
	β	−0.007	0.378** #	−0.091	0.194
**B) Bone Morphometry**					
Trabecular Area	r	0.206	−0.269** #	−0.091	−0.066
	β	0.012	−0.269* #	0.009	−0.090
**C)Trabecular Bone Micro-architecture**					
Trabecular BV/TV	r	−0.018	0.303** #	−0.084	0.172
	β	−0.010	0.377** #	−0.090	0.195
Trabecular Number	r	0.101	0.148	−0.133	0.179
	β	0.137	0.265* #	−0.117	0.190
Trabecular Thickness	r	−0.150	0.254* #	0.029	0.049
	β	−0.175	0.256* #	−0.001	0.066
Trabecular Separation	r	−0.095	−0.163	0.116	−0.162
	β	−0.149	−0.262* #	0.105	−0.176

*correlation with p-value <0.05,

**correlation with p-value <0.01,

# statistically significant after Hochberg-Benjamini correction for multiple comparison.

Abbreviations: AIS, adolescent idiopathic scoliosis; β, standardized coefficient calculated by multiple regression analysis with age and BMI adjustment; BV/TV, bone volume to tissue volume ratio; r, Pearson’s correlation coefficient; sOB-R, soluble leptin receptor; vBMD, volumetric bone mineral density.

## Discussion

This is the first study investigating the correlation between serum total leptin, sOB-R and volumetric BMD, bone morphometry and trabecular bone micro-architecture parameters as assessed by HR-pQCT for AIS subjects.

AIS girls were found to have higher sOB-R level (Table 1). sOB-R is the main binding protein for leptin in the circulation [60], [61]. Transgenic mice with over-expression of sOB-R were found to have increased circulating total leptin and enhanced leptin signal [60], [62]. High sOB-R concentrations have been shown to neutralize leptin-mediated STAT3 signaling and anorexic responses in rats [63]. sOB-R might compete with membrane anchored leptin receptor and inhibit the transport of free leptin into cells and across the blood brain barrier, thus affecting the leptin bioavailability to the cells. Based on these studies, sOB-R acts in a complex manner in that, on one hand, it could prevent circulating leptin from clearance [60], [61] and may serve as a reservoir to maintain a constant pool of readily available leptin in lean subjects [60], [64], but on the other hand, cell and animal models suggested a predominant inhibitory effect of sOB-R on leptin signaling [65], [66], [67]. In the current study, AIS patients were shown to have 10% higher sOB-R level (p = 0.006; FDR-adjusted p = 0.060) and slightly lower serum total leptin level (p = 0.493), thus indicating a higher level of bound leptin and lower level of free leptin that could result in alteration of leptin bioavailability to the target tissues.

The correlation between BMI and circulating leptin level has been well documented, and was shown to be strong and positive [47], [68], [69], [70], [71], [72], [73]. The current study showed no significant difference in serum total leptin levels between AIS and control subjects, perhaps because the subjects’ range of BMI was lower and restricted in range as compared to previous study by Qiu *et al*. [56] that included girls with BMI over 23.0 kg/m^2^. Of note, these study findings of this study are similar to our previous study [15] when comparisons are made between the same subgroup of subjects with respect to BMI.

The negative correlation between serum total leptin and sOB-R found in normal controls of this study had also been reported in the literature [73]. This phenomenon suggests the presence of a negative feedback loop with which serum total leptin could affect and regulate sOB-R level [60], [74]. This feedback loop occurs in most hormonal systems to allow precise regulation of active hormone levels [61]. The absence of this correlation in AIS could suggest cellular dysregulation in sOB-R expression or a defect in the feedback loop, thus leading to persistently high level of sOB-R in AIS.

In addition to anthropometric abnormalities and abnormal leptin bioavailability, AIS girls also have low bone mass [4], [6], [9], [16], [75], [76], [77], [78]. Previous studies by our group and others have reported that osteopenia in AIS girls is systemic in nature and could affect the whole body including the spine, hip, distal radius, distal tibia, and calcaneus [4], [6], [11], [79], [80]. This systemic low bone mass is also likely to associate with lower bone strength and peak bone mass [11], [75], [81], which might contribute to curve progression [16], osteoporosis, osteoporotic fracture, and other associated complications in late adulthood [9]. As progression of scoliosis in osteoporotic spine has been reported in adults [82], the prevention or treatment of low bone mass in patients with AIS might prevent the progression of the scoliosis as well as minimize the long term complications of osteoporosis later in life. Unfortunately, what causes the manifestation of low bone mass in AIS girls is still unknown, and it is important to reveal the underlying causes before effective means of prevention or treatment can be formulated.

Previous studies on BMD in patients with AIS were limited by the two-dimensional projection measurement of DXA for a three-dimensional structure and the resolution of pQCT was not high enough to assess important bone quality parameters including trabecular bone micro-architecture. With recent advances in bone micro-imaging technology, HR-pQCT with a resolution of up to 82 µm is now available for separate analysis of bone morphometry and vBMD in the cortical and trabecular compartments together with detailed assessment of trabecular bone micro-architecture. This new technology allows more in-depth and differential studies on bone related AIS research.

When analyzed separately, AIS girls were found to have numerically lower bone mineral status in all bone compartments with marginally significant difference detected for average vBMD and cortical vBMD. The % differences in all bone compartments were in general agreement with previous studies which reported that AIS girls had average areal BMD values 4.5% lower than controls [4], [9]. In particular, the numerically lower average vBMD found in this study was in agreement with those reported by Lee *et al*. [78] and Cheung *et al*. [6] showing lower integrated vBMD of the cortical and trabecular bones at the non-dominant distal radius and distal tibia of AIS girls. Likely due to the notably higher variance in measurement of vBMD of the metabolically active trabecular compartment [83], the difference in trabecular vBMD was not statistically significant although the % difference was similar to that of cortical vBMD (Table 2).

Leptin appears to affect bone metabolism through both central and peripheral pathways with different catabolic and anabolic effects on bone [31], [39], [84], [85]. It was suggested that there might be a balance between these two seemingly opposite effects of leptin on bone [86]. The differences in leptin bioavailability found in AIS girls versus controls (Table 1) might correspondingly affect the central and peripheral tissues differently. As suggested by a number of previous studies, the effects of leptin mediated by the CNS seemed to affect primarily cortical bone. In mice, leptin seems to regulate cortical bone formation through sympathetic activation [84], and *ob/ob* mice have diminished sympathetic tone [40]. Furthermore, β1- and β2-adrenergic receptor knock-out mice show decreased femoral mass and cortical thickness when compared to wild type mice [84]. Data from mice studies also indicate that leptin may also mediate cortical bone formation by regulating the expression of several neuropeptides in the hypothalamus. Mice lacking the neuropeptide Y receptor Y2 were shown to have increased cortical bone mass and density in the femora [87], indicating that neuropeptide Y inhibits cortical bone formation [84]. However, leptin has also been shown to increase expression of neuromedin U [88], which may decrease both cortical and trabecular bone [89]. The findings from these mice studies might serve to explain why marginal statistical significance on the difference was mainly detected for cortical vBMD but not for trabecular vBMD (Table 2).

Although the difference did not reach statistical significance for the trabecular bone micro-architecture parameters, AIS girls had numerically lower trabecular BV/TV, trabecular number, trabecular thickness, and higher trabecular separation when compared with controls. This was in agreement with findings by Lam *et al*. [79] who noted deranged bone quality as evidenced by lower Quantitative Ultrasound measurements at the non-dominant calcaneus of AIS subjects when compared with controls.

The change in bone quality in AIS is an important endophenotype. It is important to evaluate the correlation pattern of serum total leptin level and sOB-R with bone parameters which was found to be distinct in AIS subjects when compared with controls. On one hand, in girls with AIS there was a significant correlation between serum total leptin and all trabecular bone parameters (Table 4). The correlations became stronger after the adjustment for age and BMI, suggesting that the response to leptin in the trabecular bone of AIS girls is independent of age and BMI. However, on the other hand, the significant correlations between serum total leptin, sOB-R and cortical parameters became insignificant after age and BMI adjustment, suggested that the effects of age and BMI in cortical bone are much stronger than leptin and sOB-R (Table 3). It might be that the response of bone tissue to altered leptin signaling is not uniform between cortical and trabecular bone throughout the skeleton [90]. The significant correlation between serum total leptin and the trabecular parameters that was seen in AIS was not apparent in controls, thus potentially indicating an abnormal response to leptin or leptin signaling in the trabecular compartment in AIS. Trabecular bone has a much larger surface to volume ratio and is metabolically more active when compared with cortical bone [83]. This, when coupled with our findings that the trabecular area in AIS was greater than controls, the statistically significant correlation between serum total leptin level and the trabecular parameters might be expected.

The evidence of leptin effects on bone metabolism remain controversial, and together with the added uncertainty of abnormal leptin bioavailability under diseased status, careful consideration is needed during the interpretation of the discrepancy in correlation pattern of serum total leptin/sOB-R levels with bone quality parameters in our study. The possible effects of the abnormal leptin bioavailability on cells and bone metabolism are speculations based on previous findings. A scoliotic animal model would be required to provide causal evidences between abnormal leptin bioavailability/sensitivity and AIS. As mentioned earlier, AIS was associated with low bone mass which was correlated with curve severity and has been shown to be a significant prognostic factor for curve progression in AIS [16], [78]. The role of leptin and sOB-R on bone metabolism could potentially be the key for better understanding the origin of low bone mass in AIS. Research in this area can potentially lead to novel preventive or therapeutic measures for treating low bone mass and related curve progression in AIS subjects.

In summary, this is an exploratory study comparing the relationship between serum total leptin, sOB-R and cortical and trabecular vBMDs, bone morphometry and trabecular bone micro-architecture in young girls affected by AIS and normal controls. Correlation pattern between HR-pQCT bone parameters and serum total leptin and sOB-R were distinctly different between AIS and control subjects. The implication of these findings, whether there was increased response of trabecular bone to changes in the biochemical milieu in AIS and/or whether altered leptin bioavailability plays a role in the etiopathogenesis of AIS and its accompanying osteopenia warrant further studies.
